# Biological Evaluation of *Garcinia kola* Heckel

**DOI:** 10.1155/2022/3837965

**Published:** 2022-04-28

**Authors:** Abdulrahman Mahmoud Dogara, Saber W. Hamad, Harmand A. Hama, Sarwan W. Bradosty, Soran Kayfi, Sawsan S. Al-Rawi, Abubakar Abdullahi Lema

**Affiliations:** ^1^Department of Biology, Faculty of Education, Tishk International University-Erbil, Kurdistan Region, Iraq; ^2^Department of Field Crops, College of Agricultural Engineering Sciences, Salahaddin University-Erbil, Kurdistan Region, Iraq; ^3^Department of Community Health, College of Health Technology, Cihan University-Erbil, Kurdistan Region, Iraq; ^4^Medical Analysis Department, Faculty of Applied Science, Tishk International University-Erbil, Kurdistan Region, Iraq; ^5^Biological Sciences Department, College of Natural and Applied Sciences, Al-Qalam University Katsina, Katsina State, Nigeria

## Abstract

*Garcinia kola* belongs to the *Garcinia* genus of the Clusiaceae family and Malpighiales order. It contains more than 180 members all over the globe. It is found all over Asia and in tropical African countries. In Africa, traditionally, *G kola* is used to manage and treat cancer, diabetes, malaria, analgesics, hypertension, and other numerous ailments. This review aimed to comprehensively update relevant information regarding the pharmacological potential of *Garcinia kola*. Electronic databases such as ScienceDirect, PubMed, Wiley, Google Scholar, Hindawi, and Springer extracted valuable information from original scientific research papers. *Inclusion Criteria*. Antioxidant, antimicrobial, antidiabetic, antibacterial, medications, antiviral, traditional medicine, ethnopharmacology, toxicity, cytotoxic action, chemical composition, mineral elements, GCMS analysis, and any other related phrases were used as filters to find studies. *Exclusion Criteria*. Data from questionable online sources, as well as thesis reports and review publications, were excluded from this investigation. The investigation revealed that seeds of *G*. *kola* are very efficient as antioxidant, antimicrobial, antidiabetic, antihypertension, antianalgesic, and anti-inflammatory. The study also found that too much consumption of the seeds caused low fertility and toxicity. However, the safety and efficacy of *G*. *kola* have not been wholly assessed in humans, and further well-designed clinical trials are needed to corroborate preclinical findings. The mechanism of action of the seed extract should be examined. The standard dose and safety of the seed should be established.

## 1. Introduction

Traditional medicines produced from plants have become more important as alternative medicines in treating a broad spectrum of ailments, and researchers are continuing to pay attention to the use of plant materials in the treatment of many afflictions [1, 2]. The majority of the developing world believes that these plant-based products are safer and more cost-effective [3]. With the emergence of new diseases and microorganism resistance, the usage of these plant products has increased in developed, developing, and underdeveloped countries [4, 5]. Ethnopharmacology and medication discovery employing plant-based products are still critical in healthcare delivery worldwide. *Garcinia kola* is regarded as a miracle plant because every component has medicinal use. The following reviews aimed to update and do a comprehensive review regarding the biological potential of *G*. *kola*.

## 2. Methodology

Electronic databases such as ScienceDirect, PubMed, Wiley, Google Scholar, Hindawi, and Springer extracted valuable information from original scientific research papers. Inclusion criteria: Antioxidant, antimicrobial, antidiabetic, antibacterial, medications, antiviral, traditional medicine, ethnopharmacology, toxicity, cytotoxic action, chemical composition, mineral elements, GCMS analysis, and any other related phrases were used as filters to find studies. Exclusion criteria: Data from questionable online sources, as well as thesis reports and review publications, were excluded from this investigation.

## 3. Results and Discussion

### 3.1. Taxonomy, Distribution, and Morphology of *Garcinia kola*

**Figure 1:**
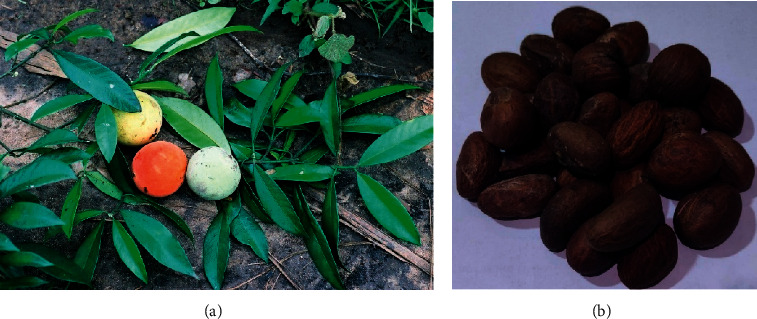
Leaf and fruits (a) [7] and seeds (b) of *Garcinia kola*.

The *Garcinia* genus includes *Garcinia kola* from the Clusiaceae family and Malpighiales order [6]. It contains more than 180 members all over the globe. Synonym names are; *Garcinia akawaensis* Spirlet, *Garcinia giadidii* De Wild, and *Garcinia bergheana* Spirlet. *G*. *kola* is a sub-Saharan African forest tree that has been dubbed a “wonder plant” since practically every portion of it has been proven to have medicinal value. It grows natively from Sierra Leone to Southern Nigeria, then on to Zaire and Angola, but it has been widely spread by man and is frequently found growing near communities. It is a tree grown in the Central and West Africa coastal rain forests. It is found all over Asia and in tropical African countries. It reaches a height of about 30 m. The orange-sized fruit is smooth and reddish yellow, with peach-like skin and yellow flesh, and three or four seeds with a brown seed coat. The seed is a nut that may be eaten. The seed coat is dark with branching lines, while the kernels are pale and punctured with resin pockets (Figure 1). Fruits are yellow, reddish, and orange-sized, with a yellow-orange, sometimes reddish pulp. The greenish-white flowers have a reddish indumentum [6].

### 3.2. Biological Evaluation

Alternative medicine is based on medicinal plants, which has led to the development of many novel pharmaceuticals [8]. More than 80% of medicine was derived from plants in the nineteenth century. The scientific revolution led to the development of the pharmaceutical business, where manufactured pharmaceuticals became more prominent [9]. There is greater usage of medicinal plants in treating ailments because they are regarded as safe and effective pharmaceuticals, have fewer side effects, and cost less than other drugs [10]. *Garcinia kola* was subjected to several biological tests (Table 1).

### 3.3. Antioxidant

The presence of free radicals and reactive oxygen species refers to oxidative stress, which is produced under normal human physiological activity but are harmful when not removed [11]. Kolaviron appears to be as effective as BHA as an *in vivo* natural antioxidant and an effective hepatoprotective agent in the current study [12]. These data suggested that *G*. *kola* seed*s* might be beneficial in minimizing the oxidative damage caused by chronic ethanol therapy in the livers of Wistar rats. The phenolic content of the antioxidant was found to be between 10 and 21 mg·g−^1^, and the scavenging was found to be between 26% and 55%, indicating that it will serve as a reservoir of natural antioxidants and be used as food enhancers [13]. Using the radical trapping test and the ion conversion method, we revealed that Ci 50 (65.86–1.17 g/mL) and the reducing power of the Ferric ion (125.4–4.91 mg/mL) are statistically significant [14]. There was a substantial increase in total white blood cell count but not in hemoglobin (*p* > 0.05). These data suggest that the seeds have immune-stimulatory capabilities, which could support the claims of ethnomedicinal efficacy (Table 1). All antioxidant biological evaluations carried out were found to exhibit significant activity irrespective of the method (Table 1).

### 3.4. Antibacterial

Consistent use of synthetic antibiotics is the leading cause of resistance in bacteria, which can be connected with biological phenomena such as membrane permeability, mutations, physiochemical changes, and efflux dynamics in target microorganisms [15]. In comparison to other microbes, bacterial strains have the genetic potential to rapidly acquire and transfer resistance to routinely used antibiotics [15]. Antibacterial medication resistance is becoming a critical global problem, prompting researchers to look for novel compounds with antibacterial properties and the potential to be used as raw materials in developing new treatments [16]. Some bacterial strains were isolated from tooth caries; therefore, the fraction of ethyl acetate hexane had the highest inhibitory activity against *Streptococcus viridans* and *Streptococcus mutans* at 0.33 and 0.33 mg·mL^−1^, respectively [17]. It is commonly used to treat toothache and prevent dental cavities, proving the traditional herbalist's claim [17]. The extracts showed an inhibitory effect on the test isolates, likely due to the high tannin and flavonoid content (Table 1). The test strains were shown to have antibacterial activity. The highest spectrum activity was seen against *S*. *mutans* and *Bacillus subtilis* at a low dosage of 1.25 mg/mL. (l4&l2 mm) [18]. Above all, our research indicates that the seed possessed antimicrobial properties. According to the findings, consuming the seed in a controlled manner may help to prevent bacterial infections in the intestine (Table 1). According to the review, the antibacterial potentials of plant extracts have been widely investigated (Table 1). The ethnobotanical research showing the traditional therapeutic potential of plant parts were confirmed in this review. According to the reported research in the following studies, all extracts tested against the tested bacterial strains, whether from human, animal, or other sources, strongly inhibited growth at a high inhibition zone (Table 1).

### 3.5. Antifungal

The utilization of plant extracts as sources for developing novel antifungal medicines has long been practiced. Plant-based medicines have considerably enhanced human health and well-being. The extracts also had antifungal efficacy against *Aspergillus niger*. Compared to the standard antibiotics used in the investigation, the data show that the compound has substantial antifungal properties [19]. In a fungistatic approach, the seed extract exhibits high action against *Candida albicans* and *Aspergillus flavus* (Table 1). The MICs of ketoconazole [standard medications], which had a range of 275–691/mL and 346–318/mL, respectively, the fungus ranged from 275–691/mL and 346–318/mL. These findings suggest that the extract may include compounds that can combat microbial illness [20].

### 3.6. Antiviral

This research has found that the extract's ability to immediately remedy a patient's ocular symptoms and indicators is obvious and encouraging (Table 1). Given the lack of a particular antiadenoviral medication on the market, this could be a game-changer in treating these viral infections [21]. According to this study, *G kola* is effective against viral infection and in areas where resources are scarce (Table 1).

### 3.7. Antihypertension

Hypertension, well-known as high blood pressure, is considered by persistently excessive blood pressure in the arteries [22]. High blood pressure can damage arteries supplying blood to the kidneys, heart, brain, and eyes [22]. The blood pressure of rats fed *G kola* enriched meals dropped significantly by the third week at *p* < 0.05. Finally, *G*. *kola* contains a vasoactive component that can reduce blood pressure. However, the actual method of action is still unknown (Table 1). Traditional medicinal practitioners have always advocated for using *G*. *kola* parts to treat hypertension. The findings of the following studies bring up new research options for new antihypertensive drugs or herbal formulations. Plant-based treatments are considered effective.

### 3.8. Anti-Inflammatory

Inflammation is the body's natural response to damage or foreign irritation. Inflammation, marked by pain, has been known to humanity since the dawn of time. Since the dawn of time, humans have been looking for ways to reduce and manage inflammation, including using plants [1]. Treatment with 25, 50, and 100 g/mL inhibited cell proliferation in a dosage and time-dependent approach. The inclusion of chemicals with anti-inflammatory characteristics contributed to the study's findings [23]. It could be beneficial in conditions marked by cellular proliferation and inflammatory reactions [24].

### 3.9. Antidiabetic

Diabetes mellitus is a metabolic condition characterized by hyperglycemia, the most prevalent symptom. Its chronic stage impacts blood vessels, kidneys, the heart, and nerves [25]. Diabetes affects 463 million people worldwide, and that number is expected to rise to 578 million by 2030 [25]. At a dose of 100 mg·kg^1^, kolaviron linked bioflavonoids effectively reduced hypoglycemic symptoms in normal and alloxan diabetic rabbits (Table 1). Compared to the controls, there was no significant change (*p* > 0.05) in single-dose glucose levels, long-term HDL levels, or body weight. However, glucose (mmol/L) levels in the four-week treated rats were significantly lower (16.22.9; *p* > 00.05) than in the controls (21.63.6), and LDL levels were 66% lower in the treated group (*p* < 0.01; 86.818.2 against 29.810.9) (Table 1). On day 7, the 500 mg/kg ethanolic seeds extract-treated group had a 49.70% drop in blood glucose levels compared to the positive control group (45.03%). The findings of this investigation suggested that the seed could be used to treat illnesses and diabetic management [26]. The results mentioned above validate the usage of the plants in the traditional medicinal system to treat diabetes by traditional practitioners.

### 3.10. Antianalgesic

Controlling acute and chronic pain has become a serious concern, particularly among the elderly. Pain is a nonspecific symptom of many diseases that lead to unpleasant emotional and sensory experiences. The findings show that the chemical possesses dose-dependent antinociceptive properties against acetic acid-induced abdominal constriction in mice (Table 1). At all doses, there was a reduction in the number of writhes compared to control animals at *p* < 0.05. The seed has antianalgesic properties [27]. The studies examined in the following study found the extract from bitter kola exhibited strong antianalgesic properties.

### 3.11. Antipneumonia

Pneumonia is an inflammatory, infectious lung disease condition that affects the mucosal parts of the lungs and can be acute and persistent [28]. Fungi, bacteria, and viruses cause the disorder. Anti-*Klebsiella pneumonia* activity rose when kolaviron concentrations dropped. Kolaviron was efficacious at 500 mg/kg and showed a significant difference at *p* < 0.0001. Bitter kola can treat pneumonia because it contains antimicrobial properties (Table 1).

### 3.12. Antiobesity

Obesity is a complicated health condition classified as a chronic disease that has a detrimental impact on the human body [29]. Obesity raises the risk of diabetes, hypertension, heart disease, and other serious illnesses. Obesity cases are increasing at an alarming rate worldwide [30]. There are currently more than 300 million obese people on the planet [31]. The results revealed a considerable rise in the counts of RBCS in both tested animals, as well as a reduction in their weight. Very low-level density of lipoprotein in the plasma was reduced in the approach of dependent dose, while the level of chylomicrons increased in a dependent-dose approach. Low levels of high-density lipoproteins and an increase in low-density lipoproteins play a role in cardiovascular diseases (Table 1).

### 3.13. Fertility Evaluation

Medicinal plants have long been used to boost or manage fertility. The experimental model was divided into three groups: groups 1 and 2 received the extracts orally at doses of 400 and 200 mg for 28 days, respectively, while group 3 served as a control group. According to the study, group 1 had slight interstitial congestion disorientation of the cells, whereas group 2 had a normal interstitial space with germinal epithelium regeneration and a small number of matured spermatozoa. As a result, this study suggests that a high-calorie diet could have a deleterious impact on sperm parameters and testis shape [32]. This discovery demonstrated that bitter kola could reduce fertility in male Wistar rats [33]. The extract has been proven to have an antispermatogenic effect. It can damage the male reproductive organs, necessitating controlling the amount consumed (Table 1).

### 3.14. Antiglaucoma

Everywhere across the globe, glaucoma is the most common cause of permanent blindness [34]. The most prevalent kind of primary open-angle glaucoma (POAG) is characterized by progressive optic nerve degeneration and affects over 60 million individuals worldwide. In the African continent, 15% of blindness was due to glaucoma [34]. After taking it orally, healthy young people's intraocular pressure was lowered by 21%. In low-income settings, patients with POAG or ocular hypertension may benefit from such an effect (Table 1).

### 3.15. Antitrypanosome

Humans and animals are both affected by trypanosomiasis, a parasite disease. Trypanosoma is a parasite species that causes the disease. More than 50 million individuals and more than 50 million animals are infected worldwide [35]. Only the experimental model that received the dose of 600 mg/kg per day of their body weight, which got a very minimal parasite total for nearly four months after therapy, was terminated. Yet, all those who were on it died (Table 1).

### 3.16. Ingestion

The results revealed that the erythrocyte count, PCV, and hemoglobin concentration had all dropped significantly. When evaluated on mammalian erythrocytes, this shows that the active component has no long-term toxicological effects (Table 1).

### 3.17. Geotactic Behavior

All living species have an inbuilt behavioral response called geotaxis, defined by motor actions toward or away from the Earth. Flying animals, in particular, have a lot of negative geotaxis against Earth's gravity [36]. In flies fed a diet enriched with higher *G*. *kola* seed inclusions, GST, and catalase activities were dramatically boosted, whereas no content was significantly reduced compared to controls (Table 1).

### 3.18. Steroid Hormones

These data imply that the seed extract plays a function in cortisol, potassium, and sodium secretion regulation (Table 1). Despite its potential benefits, it should be used with caution because it is a depressive drug [37]. These data imply that plays a function in cortisol, potassium, and sodium secretion regulation. It should be used with caution because it is a depressant (Table 1).

### 3.19. Growth Performance

The moisture, protein, and ash content of the fish carcasses did not differ across the treatments (*p* > 0.05). The data suggest that feeding *G*. *kola* seed powder *to Clarias gariepinus* fingerlings boosted growth rate, feed utilization, and survival (Table 1). At *p* > 0.05, there were significant variations in the growth metrics and the food conversion ratio. Compared to the other treatments, the fish given 1.0 g/kg ethanolic seed extract diets gain the most weight. This supports the plant's probiotic advantages as a growth promoter (Table 1).

### 3.20. Healing of Liver Injury

The liver is a vital organ in our body responsible for most metabolic and secretory functions. As a result, it appears to be a sensitive target for drugs that modulate biotransformation [38]. The duration or persistence of a liver injury is arbitrarily split into acute and chronic liver injury in clinical practice [39]. The researchers discovered that combining the two plants had a therapeutic effect on the healing of the injured liver. This backed up its long-standing usage in treating individuals with liver infections (Table 1). The plant has the potential to be utilized in the development of drugs for liver treatment.

### 3.21. Hematological Analysis

As a result, the aqueous seed extract has a minimal erythropoietic effect but causes moderate leucopenia with lymphocytosis and a decrease in all other WBC lines (Table 1). The extract significantly decreased the volume of the cell mean cell and hemoglobin cell means in the plasma of the animals (*p* < 0.05). The ethanolic extract of *G kola* seed has hematological, stimulating, and enhancing effects due to its antioxidant qualities [40]. These findings suggest that it has no harmful effects on the liver's function and may have a beneficial effect, as indicated by its capacity to drastically lower total serum cholesterol and increase WBC count [41].

### 3.22. Cytotoxicity

Many plant-derived chemicals have now been shown to have antibacterial, anticancer, and other biological properties [129]. Parts of medicinal plants are considered the reservoir of a novel compound with a therapeutic potential to treat a wide array of diseases compared to the synthetic drugs available [130]. Many studies have proven that medicinal plants contain a wide array of compounds that have a positive biological effect [8, 11]. These components are only beneficial if they are confirmed to be nontoxic or have minimal toxicity. Quite a number of studies have been carried out on the toxicity of *G*. *kola* parts (Table 1) both *in vivo* and *in vitro*. Higher dietary intake of *G*. *kola* seeds drastically lowered the survival rate of *D*. *melanogaster* compared to control flies [42]. These findings could be linked to the bioactivity of *G*. *kola* seed components such saponins and glycosides, both of which are hazardous in large doses. The extract did not appear to have any substantial toxicological effects on erythrocytes, although it did tend to increase erythrocyte amount over time [127]. The results showed that neither medicinal plant extract had any significant negative effects on total protein or glutamate pyruvic transaminase at *p* > 0.05 compared to the control [86]. *Garcinia kola* has modest toxicity, with an oral 50% fatal dose of over 5000 mg/kg bw [52]. Based on the study's findings, excessive usage of *G*. *kola* seeds may have toxicological implications, and moderate use is consequently recommended.

### 3.23. Chemical Compounds Responsible for the Biological Activity

**Figure 2:**
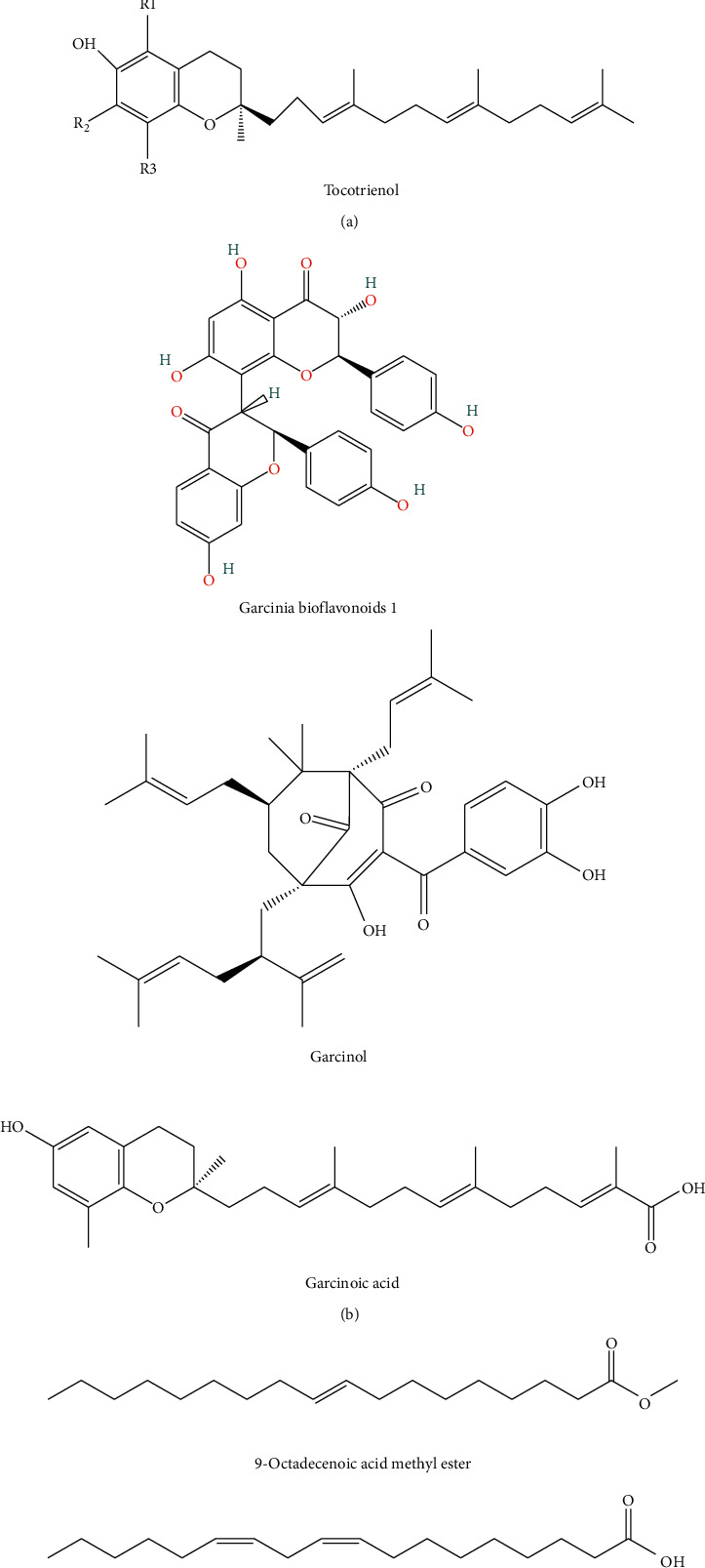
Some of the chemical structures found in *G. kola* are responsible for its biological activity.

Due to the presence of tannin in the plant, it could be used to cure burns and wounds [131]. The plant's high alkaloid and flavonoid content suggest that they have antioxidant potential and explain their medicinal activities, which might be exploited in drug formulation [131]. The presence of large levels of flavonoids in all plant parts demonstrated that the plants perform biological tasks such as protecting against allergies, free radicals, microbes, ulcers, inflammation, hepatotoxins, and viruses (Figure 2). Natural compounds, including garcinoic acid, garcinol, and tocotrienol extracted from the seed of *G*. *kola* from Nigeria, have 1.5 times the antioxidant activity of a-tocopherol [52]. The ME4 fraction was chromatographically fractionated and spectroscopically analyzed, revealing the presence of some compounds: Garcinia biflavonoids 1, Garcinol and Garcinoic acid (Figure 2). These findings suggest that these four chemicals are responsible for some of *G*. *kola* seeds' high antioxidant activity. This adds to the evidence of *G*. *kola*'s nutraceutical and medicinal potentials [132]. The ability of a plant extract to inhibit bacteria, particularly those with substantial health implications, is mainly dependent on essential phytochemical components having antimicrobial activity [53]. The presence of a wide range of chemicals in extracts from various plant sections has been linked to their pharmacological properties [53]. The following compounds were reported present in the essential oil extracted from the seed 9-Octadecenoic acid methyl ester, 9,12-Octadecadienoic acid (Z, Z), Stearic acid methyl ester, and Hexadecanoic acid methyl ester; they are reported to be responsible for antibacterial, antioxidant, and many more pharmacological properties (Figure 2). Research uncovered *G*. *kola* was discovered to possess numerous chemical components that have antioxidant properties [133]. Benzophenones, flavonoids, and xanthenes are among the components found in *G*. *kola* (Figure 2). They are known to have antiparasitic, anti-inflammation, antibacterial, and antiviral activities [110]. The anti-inflammatory action of the seed is considered due to the presence of flavonoids and benzophenone [134].

## 4. Conclusion and Future Recommendations

Research into the pharmacological benefits of medicinal plants provides us with critical knowledge for better organizing current and future studies to address a variety of human illnesses. *G. kola* is a remarkable medicinal plant with a variety of traditional usage that has been documented since antiquity. Preclinical investigations have already been conducted on a variety of biological activities. The seeds were found to have significant biological activity, and this is due to the *G*. *kola* containing nutritionally and pharmacologically essential compounds. Research into the mechanisms behind the bioactivity of the constituent chemical components is required. As a result, well-designed clinical trials are recommended to obtain more conclusive evidence about the usefulness of *G*. *kola* seeds.

## Figures and Tables

**Table 1:** Biological evaluation.

S/N	Activity	Method		Extract	Major findings	Reference
1	Antioxidant	*In vivo*	Seeds		In flies fed a diet enriched with higher *G*. *kola* seed inclusions, GST and catalase activities were dramatically boosted, whereas NO content was significantly reduced compared to controls.	[42]
		*In vivo*	Seed	Petroleum ether	Kolaviron appears to operate as an in vivo natural antioxidant and an effective hepatoprotective agent in the current investigation. It is as effective as BHA.	[12]
		*In vivo*	Seeds	Ethanol	These findings showed that seeds could be useful by reducing the oxidative damage produced by chronic ethanol treatment in Wistar rats' livers.	[43]
		*In vivo*	Seed	Methanol	Significant rise in total white blood cell count with no increase in hemoglobin at *p* > 0.05. These findings imply that the seeds have immune-stimulatory properties, which could support ethno-medicinal efficacy claims.	[44]
		DPPH, FRAP	Seed	Ethanol and aqueous	Using radical trapping test and the ion conversion method revealed that Ci 50 (65.86, 1.17 g/mL) and the reducing power of the ferric ion (125. 4 4. 91 mg/mL) are statistically significant.	[14]
		DPPH	Seed/EO	Hexane	The highest scavenging activity was recorded at 85.6%.	[45]
		DPPH	Seeds	Methanol, chloroform, and ethyl acetate	Antioxidant activity through radical scavenging activity was found to be 46.00. In terms of antioxidant activity, the total polyphenols showed a significant level of association (r2 = 0.927).	[18]
		DPPH, FRAP and FTC	Seeds	Petroleum ether, acetone, and ethanol	The results of the three test methods revealed that all extracts, regardless of the solvent employed for extraction, had strong antioxidant activity starting at 0.5 mg/mL.	[46]
			Seed	Ethanol	This discovery suggested that the extracts may include antioxidants and hence can scavenge free radicals, thereby preventing oxidative stress. This may support their use in treating hepatic dysfunction and stress-related disorders on a local level.	[47]
		*In vivo* and *in vitro*	Root		According to the biological evaluation, the saponin extract from the root has scavenging actions against free radicals. The root has the potential to be used as a natural antioxidant source.	[48]
			Seeds	Methanol	The ME4 had the highest level of activity. The ME4 fraction was also significantly reduced nitric oxide generation in lipopolysaccharide-activated macrophage U937 cells.	[49]
		*In vivo*	Leaf	Cold 70% ethanol	The extract inhibits the most in both liver and brain homogenates at the same concentration (26.7 g/mL), with the percent inhibition of 64.1% and 38.25, respectively.	
		DPPH, FRAP and Fe^2+^ chelating	Seed	Aqueous and ethanol	At *p* > 0.05, the ethanolic extract had considerably better characteristics. Given this, the usage of the plant in traditional medicines for the treatment of cough and liver diseases could be linked to its phytochemical composition.	[50]
		DPPH, FRAP	Seeds	Aqueous	It exhibited significant antioxidant activity at varying doses, which might be attributed to diverse phenolic components in the plants.	[51]
		*In Vivo*	Seeds		Compared to the control group, prolonged administration had no negative effects on spermatozoa features but considerably increased testosterone concentration. Malondialdehyde levels in the liver, testes, and spermatozoa of rats were much lower as antioxidant systems improved. When compared to controls, prolonged administration of *G*. *kola* had no effect on the liver and testes at all doses, according to histological analysis.	[52]
					The antioxidant regarding the phenolic content was found between 10–21 mg·g^−1^. The scavenging at 26%–55% was high, showing that it could be a good source of natural antioxidants and employed as food supplements.	[13]
		DPPH	Seed oil	n-hexane	The highest scavenging activity was recorded at 91.05 ± 0.12 mg/mL.	[53]
		DPPH	Seed	Ethanol	The antioxidant studies revealed a dose-dependent substantial (*p* > 0.05) increase in its ability to scavenge free radicals. The findings of this investigation suggested that the seed could be used to treat free radical-mediated illnesses.	[26]
		*In vivo*	Seed	Ethanol	On day 7, the 500 mg/kg extract-treated group had a 49.70% drop in blood glucose levels compared to the positive control group (45.03%). The findings of this investigation suggested that the seed could be used to treat illnesses and diabetic management.	[26]
		Linoleic acid system	Seed	Petroleum ether	Seeds overall antioxidant activity on lipid peroxidation might be ascribed to their ability to scavenge free radicals and active oxygen species. It could be linked to the inhibition of *in vivo* lipid peroxidation propagation.	[54]
		*In vivo*	Seed	Ethanol	When compared to rats in group 2, the glutathione concentration of the group was significantly lower (*p* < 0.05). Compared to rats in group 1 and the treatment group, the vitamin C level in group 2 was significantly lower (*p* < 0.05).	[55]
2		Agar well diffusion	Seeds	Acetone	The synergistic efficacy of bitter kola and fantastic kola exhibited superior antibacterial activities. The positive results for both Gram negative (*Escherichia coli*, *Pseudomonas* sp.) and Gram-positive (*S*. *aureus* and *Bacillus* sp.) bacteria indicate that they could be employed as broad-spectrum antibiotics.	[56]
		Agar well diffusion		Ethan, aqueous	At a 30 mg/mL of ethanol and aqueous (hot water) dosage, extracts showed higher antibacterial activity, with zones of inhibition ranging from 17 to 23 mm for ethanol.	[19]
		Agar dilution method	Seed	Methanol and aqueous	This study found that *G*. *kola* extracts have good antifungal activity against clinical isolates of *Fusobacterium nucleatum* and its connection with periodontal infections.	[57]
			Seed	Ethanol	The extract was the most effective against the test organisms, with a mean inhibition zone of 15.33 mm. As a result, it can be deduced that bitter kola, kola nut, and avocado seeds exhibit antibacterial action, with the kind of extracting solvent having a significant impact on the level of antimicrobial activity. This means that an antibacterial seed or herb extract should be made in the most appropriate solvent for maximum efficiency.	[58]
		Disc diffusion method	Leaf	Methanol and aqueous	The extracts ranged from 25 mgL^−1^ to 50 mgL^−1^. The findings suggest that these plants' leaves could be utilized to treat ailments caused by the test organisms. The bioactive components of the leaves would be characterised further using crude extracts.	[59]
		Agar-well diffusion method	Seed	Methanol	At a 20 mg/mL final dosage, the extract showed considerable inhibitory effect against all examined bacteria except four. The inhibition zones varied from 10 to 23 mm, while the typical antibiotics' zones of inhibition ranged from 15 to 25 mm; 12 and 25 mm, respectively.	[60]
		Tube dilution susceptibility	Leaves (combine with other plants)	Aqueous	The findings imply that the formulation has high in vitro antibacterial activity against common wound isolates and could be used for routine wound and sepsis treatment instead of antibiotic chemotherapy.	[61]
		*In Vivo*	Seed	n-hexane, hot aqueous and ethanol	The best antibacterial activity was found in the n-hexane extract, followed by ethanol and finally hot water. According to MIC, the inhibitory zone diameter of n-hexane clove extract was the biggest, followed by bitter kola extract, and finally tobacco extract.	[62]
		Test tubes bottles	Seed oil	n-hexane	The oil was discovered to have broad-spectrum activity against gram-positive and gram-negative bacteria isolates, which was concentration-dependent.	[63]
		Agar-well diffusion method	Seeds	Methanol, aqueous	At the same dose of 2.5 mg/mL, the extract had bactericidal activity against *Klebsiella pneumoniae* and *Shigella* species. As a result, pharmaceutical companies should examine extracts that have been demonstrated to be effective against test organisms.	[64]
		*In vivo*	Seed	Aqueous	The findings show that both uncoated and coated bitter cola has medical promise as a lead toxicity reducer and alternative antibacterial. Furthermore, it could be a two-edged sword for treating lead toxicity and subsequent infections caused by lead poisoning.	[15]
		Microdilution broth method	Leaf (EO)		The oil contains several chemicals that were active against the bacteria tested, with minimum inhibitory concentrations ranging from 50 to 400 g/mL and might be used to produce plant-based medications.	[65]
		Agar diffusion method	Seeds	Petroleum ether, 70% ethanol and aqueous	The presence of a polyisoprenyl benzophenone (Kolanone) in the petroleum ether extract and the hydroxy biflavanonols in the ethyl acetate fraction was found to be responsible for the observed activity.	[66]
		MIC	Seed	Ethanol	The extract had a broad spectrum of activity, whereas the fractions had a narrow spectrum of activity because they were only active against *S*. *aureus*, *E*. *coli*, and *Pseudomonas aeruginosa*. These findings could explain why *G*. *kola* seeds are useful in treating microbial diseases.	[67]
				Methanol	Compared to chloramphenicol [standard medicine], which had MICs of 14.31–31.62 g/mL, the minimum inhibitory concentration (MIC) for bacteria.	[20]
		Agar disc diffusion method	Seed	Aqueous	As a result, the findings imply that biogenic AgNPs have potential biological applications and might potentially be used as a key component in the development of innovative nanopaints against the tested bacterial strains.	[68]
		Disc diffusion	Seed	Aqueous, ethanol and methanol	This study reveals that seeds extracts from these plants have antibacterial characteristics and could be utilized as an alternative to antibiotics.	[69]
		Checkerboard technique	Seeds	Methanol	The extract's MICs against microorganisms were found to be 1.562 and 3.125 mg/mL, respectively.	[70]
		Disk	Seeds		The ethanol seeds extract was found to have significantly higher activity (*p* < 0.001) than the aqueous preparation. The presence of several pharmacokinetic substances could explain the activity.	[71]
		Agar-well diffusion method	Seeds (black nanocrystal of silver nanoparticles)	Aqueous	All of the bacteria examined showed that the produced silver had good antibacterial action. Green nanoparticles can be employed in a variety of medicinal applications.	[72]
		Agar diffusion method	Seed	Methanol and aqueous	There was a higher level of activity with the hot water seeds extract. The findings supported herbalists' historical usage of botanicals in treating bacterial illnesses.	[73]
		Agar-well diffusion method	Seed	Petroleum ether	Antimicrobial activity against a broad spectrum of microorganisms has been observed in the isolated chemical.	[74]
		Disc	Seeds	Aqueous and ethanol	At *p* < 0.05, the results were significant. Against the bacterial isolates, the extracts demonstrated different levels of inhibition.	[75]
		Disk	Seeds	Aqueous and ethanol	At *p* < 0.05, the results were significant. Against the fungal isolates, the extracts demonstrated different levels of inhibition.	[75]
		Agar-well diffusion method	Leaves	Cold aqueous, hot aqueous, ethanol and methanol	The MIC was evaluated at different concentrations of 25 and 12.5 mg/mL and showed efficacy. The findings support the plant's long-standing use in Nigerian rural communities to treat infectious disorders.	[76]
		Agar well diffusion	Seeds	Methanol, chloroform, and ethyl acetate	Antibacterial activity was found in the plant seed extracts against the test strains. However, at a low dose of 1.25 mg/mL, the maximum spectrum activity was observed against *S. mutans* and *B. subtilis* (l4 and l2 mm). The findings suggested that using these plant extracts as nutraceuticals could help to minimize the risk of microbial infections.	[18]
		Agar well diffusion	Seeds	100% (neat)	With a zone diameter of 22.0 mm and above, the extract showed significant inhibition against the strains.	[77]
			Seeds	Methanol	Except for *E*. *coli*, all studied bacterial strains have an inhibition zone diameter of roughly 20 0.91 mm. At *p* > 0.05, the standard used (tetracycline) had a larger zone of inhibition. Antibacterial characteristics were present in the extract.	[78]
		Fractional inhibitory concentration	Seeds	Acetone	Combinations against gram-positive species yielded mostly synergistic interactions (FIC index of 0.52—0.875), while combinations against gram-negatives yielded more antagonistic interactions (FIC indices of 2.0–5.0). We infer that the seed extract could be a source of broad-spectrum antibiotic resistance-modifying chemicals.	[79]
		Disc diffusion	Seed	Ethanol (70%)	The extracts showed an inhibitory effect on the test isolates, likely due to the high tannin and flavonoid content. Above all, our research indicates that the seed possessed antimicrobial properties. According to the findings, consuming the seed in a controlled manner may help to prevent bacterial infections in the intestine.	[16]
		Agar well diffusion	Seed	Ethanol and aqueous	The effects of various concentrations were studied. It was discovered that a synergistic blend of aqueous and honey seed extracts was more effective than using the extracts separately in suppressing the growth of the bacterial strain.	[80]
		Disc	Seeds	Petroleum ether, acetone and ethanol	Antibacterial sensitivity testing revealed that the extracts reduced the growth of the test isolates, as evidenced by measured zones of inhibition, which differed between species.	[46]
		Ager well	Seeds	Ethyl acetate, ethanol, methanol, acetone and aqueous	The extracts had inhibitory zone widths ranging from 0–24 1.1 mm, with MIC and MBC values of 0.04–1.25 mg/mL and 0.081–2.5 mg/mL, respectively. The findings of this study support the use of this plant in traditional medicine and provide a lead for the creation of new and powerful antimicrobials.	[81]
		Bottles of molten agar	Seeds	Methanol	The antibacterial activity against all isolates was significantly lower than the standard antibiotic, gentamicin 4 mg/mL, at *p* > 0.05. Similarly, the activity was dose-dependent, with greater inhibition zones corresponding to higher concentrations at *p* > 0.05.	[82]
		*In vivo*	Seeds	Aqueous	At 1 and 2.5 h, the interaction was antagonistic, but at 4 h, it became potentiated. The actual mechanism that causes the observed biphasic interaction is unknown.	[83]
		Agar diffusion method	Seeds	Aqueous, ethanol, and methanol	The crude extracts' sensitivity patterns of inhibition zones revealed a proportionate degree of inhibitory activity against the tested bacterial strain.	[84]
		Agar well diffusion	Seeds	Ethanol	The findings of this investigation revealed that the extract had inhibitory activity against the bacterial isolates tested at various concentrations, with a greater inhibitory effect on *E*. *coli* at a concentration of 300 mg/mL shows that as the concentration of the extract against the bacteria increases, the zones of inhibition expand.	[85]
		Cup plate method and broth dilution methods	Seeds	Ethanol	Fraction of hexane: ethyl acetate 70 : 30 had the highest activity against *S*. *mutans* and *S*. *viridans*, with MICs of 1.50 mg·mL^−1^ and 0.33 mg·mL^−1^, respectively,	[17]
		Agar dilution method	Seeds	Ethanol and aqueous	*S*. *aureus and K*. *pneumonia* had minimal bactericidal concentrations of 1.00 mg/ml and 0.50 mg/mL, respectively.	[86]
		Disc method	Seeds	Ethanol	The plant seeds should be recommended for treating *E*. *coli* diarrhoea and all *B*. *cereus* diseases based on this research; nevertheless, more research is needed to isolate the medicinal chemicals, explore their mode of action, and the effect of the same in the *in vivo* environment.	[87]
		Disc diffusion	Seed	Aqueous, acetone, methanol and ethanol	The findings demonstrated that methanol extract has the highest inhibitory activity at various doses against all tested bacterial strains, with *S*. *aureus* having the maximum zone of inhibition (*p* > 0.05).	[88]
		Agar-well diffusion	Seed	Methanol and aqueous	It is concluded that secondary metabolites included in the extract are responsible for the bacteria inhibition reported in this investigation; consequently, the test plant could be used to make medications to treat illnesses caused by the test organisms.	[89]
			Seed	Methanol	These seeds extracts' antibacterial properties could be effective in treating multidrug-resistant *Acinetobacter baumannii*. The aqueous fraction outperformed the other fractions in terms of activity.	[90]
		Agar-well diffusion	Seeds and leaves	Aqueous, ethanol, and methanol	Both the leaves and seeds extracts had a substantial antibacterial impact on *S*. *aureus.*	[91]
		Cork-borer	Seed	Aqueous, ethanol, and methanol	On the other hand, the extracts had a stronger antibacterial activity, with a ZOI of 8.66 0.42 mm (5.30.4–13.50.4) compared to 6.36 0.36 mm (3.90.06–8.906 mm).	[92]
		Disc	Bark	Aqueous	The antibacterial screening of the biosynthesised AgNPs revealed that they had inhibitory potential and could hinder microorganisms' growth.	[93]
		Tube dilution	Leaves	Aqueous	The findings imply that the formulation has high in vitro antibacterial activity against common wound isolates and could be used for routine wound and sepsis treatment instead of antibiotics and chemotherapy.	[94]
		Agar well method	Bark and seeds	Ethanol	The extract inhibited all tested bacterial strains in a zone of inhibition ranging from 12 to 23 mm.	[95]
		Agar well diffusion			More research is needed to determine the sort of antimicrobial activity they exhibit (bactericidal or bacteriostatic), as well as the active components contained in the vinegar samples that allow them to exhibit such activities.	[96]
		Disc	Seed	Ethanol	Antibacterial activity tests revealed that all three eluates had cumulative bactericidal activity against five of the ten species tested. The pyridine/pyrimidine moiety in Eluate 2 suppressed the development of *K. aerogenes* in a way that the other eluates and the broad-spectrum antibiotic levofloxacin did not.	[97]
		Agar diffusion method	Seeds	Aqueous and ethanol	The various test plant extracts moderately inhibited the standard bacteria *E*. *coli* NCTC 10418 and *S*. *aureus* NCTC 6571, with inhibition zones ranging from 8 mm to 20 mm. The antibacterial properties of these plants are revealed in this investigation.	[98]
		Agar well diffusion method	Seeds	Methanol	The extract exhibited strong activity against the tested strains.	[99]
		Agar diffusion	Seed oil	n-hexane	Antibacterial tests revealed a high susceptibility to all germs examined. *Salmonella typhi* was the most susceptible of the bacteria tested, with an inhibition of 27 mm, while *E. coli* had the least, with an inhibition of 12 mm, at a dose of 100 mg/mL.	[53]
			Polyphenolic		IR, 1H, and 13C-NMR spectroscopy were used to characterize the fraction with the strongest antibacterial potential. The molecule could be Catechin, methyl-dl-tyrosine, p-naphtholbenzein—or Naringin, according to the combined spectroscopic data.	[100]
		Agar well diffusion	Leaves	Ethanol, methanol, hot and cold aqueous	The findings revealed that of the 96 wound swabs collected, 15 (21.7%) bacteria pathogens were identified in the following order: *E*. *coli* 9 (60%), *P. aeruginosa* 4 (26.6%), *Klebsiella* spp 1 (6.6%), and *S. aureus* 1 (6.6%).	[101]
		Agar well diffusion	Seeds	Methanol, ethanol, and aqueous	Methanolic and ethanolic seed extracts were found to have antibacterial action against gram-positive and gram-negative bacteria.	[102]
3	Antifungal	Agar well diffusion	Seeds	Methanol, ethanol, and aqueous	No activity.	[102]
		Agar well diffusion	Seeds	Ethanol and aqueous (cold and hot)	Antifungal activity was also found in the seed extracts against *A. niger*. Compared to the usual antibiotics employed in the study, the results indicate strong antifungal capabilities.	[19]
			Seeds	Methanol lead acetate	On two beer spoilage microorganisms, *Candida vini* and *Lactobacillus delbruckii*, exhibited significant activity.	[103]
		Agar diffusion method	Seeds	Petroleum ether, 70% ethanol and aqueous	The extract has significant activity against *C. albicans* and *A. flavus* in a fungistatic manner.	[66]
		MIC	Seed	Ethanol	It was also effective against fungi such as *Penicillium notatum* and *A. niger.*	[67]
		TTest tubes bottles	Seed oil	n-hexane	The oil was discovered to have broad-spectrum activity against fungal isolates examined in the following study, which was concentration-dependent.	[63]
		Agar well diffusion	Fruit mesocarp	Methanol	Ketoconazole [standard medicines], which had MICs of 2.66–2.99 g/mL, fungi ranged from 275.4 to 691/mL and from 346.7 to 318.2/mL, respectively. These findings show that the extract could be a source of chemicals that can be employed to fight microbial infection	[20]
		Microdilution broth method	Leaf (essential oil)		The oil contains several chemicals that were active against the fungi tested, with minimum inhibitory concentrations ranging from >400 to 50 g/mL and might be used to produce plant-based medications.	[65]
		In vivo	Seed	Aqueous	According to this study, seeds of *G*. *kola* are good against candida infection. In resource-constrained regions.	[104]
		Agar-well diffusion method	Seeds (black nanocrystal of silver nanoparticles)	Aqueous	The fungal examined showed that the produced silver had good antibacterial action. Green nanoparticles can be employed in a variety of medicinal applications.	[72]
		Agar Disc diffusion method	Seed	Aqueous	As a result, the findings imply that biogenic AgNPs have potential biological applications and might potentially be used as a key component in the development of innovative nanopaint against the tested fungal strains.	[68]
		Disc	Seeds		*C. albicans* had no response to various concentrations used in the water extract. The ethanol extract was found to have significantly higher activity (*p* > 0.001) than the aqueous preparation. The presence of several pharmacokinetic substances could explain the activity.	[71]
		Agar well diffusion	Seeds	Ethanol	On the fungal isolates, the extract has no inhibitory effect.	[85]
		Checkerboard assay	Seeds	Ethanol	In comparison to their separate activities, the combined activities of the two extracts demonstrated a significant improvement in anti-Candida activity. The findings suggest using the ethanolic seeds extracts' with individual bioactive ingredients and combining them to create viable antifungal medicines.	[105]
5	Antiviral	*In vivo*		Aqueous	This research has found that the extract's ability to immediately remedy the patient's ocular symptoms and indicators is obvious and encouraging.	[21]
5	Antihypertension	*In vivo*		Chloroform, methanol	In the third week, rats fed *G*. *kola* enriched diet showed a significant drop in blood pressure (*p* > 0.05). Finally, *Garcinia kola* includes a vasoactive component that can help to decrease blood pressure. However, the exact mechanism of action is still unknown.	[106]
		*In vivo*			After histaminergic blockage, however, there was a substantial (*p* < 0.05) decrease in the extract effect. According to this study, the alcohol extract of *G*. *kola* has a vasoactive component that lowers blood pressure.	[107]
		*In vivo*			The intraocular pressure of healthy young people was reduced by 21% after taking it orally. Patients with POAG or ocular hypertension in low-income settings may benefit from this effect.	[34]
6	Anti-inflammatory	*In vivo*	Seed		When compared to aspirin, the anti-inflammatory potency of acetylsalicylic acid demonstrated rather good anti-inflammatory action. The greatest edema inhibition achieved in rats pretreated with 100 mg/kg kolaviron (59.52% ± 4.65) is comparable to that obtained with 150 mg/kg Aspirin (62.05 ± %3.75).	[108]
		Cell proliferation assay	Seed		*G kola* seeds appears to have the capacity to reduce mitogen-activated vascular cell proliferation as well as inflammatory responses.	[24]
		*In vivo*	Seed	70% methanol	In albino Wistar rats, the extract at dosages of 500, 1000, and 1500 mg/kg exhibited a statistically significant (*p* < 0.01) dose-dependent decrease of brewer's yeast-induced pyrexia. The findings reveal that the extracts had a strong antipyretic effect, indicating that their ethnomedicinal use is justified.	[109]
		*In vivo*	Seeds		*G*. *kola* seeds appeared to provide clinically significant analgesic/anti-inflammatory benefits in knee osteoarthritis patients.	[110]
		MTT assay	Seeds	Methanol	Treatment with 25, 50, and 100 g/mL inhibited cell proliferation in a dose- and time-dependent manner. The inclusion of chemicals with anti-inflammatory characteristics contributed to the study's findings	[23]
7	Antidiabetic	*In vivo*	Seeds	Ethanol	Compared to the controls, there was no significant difference (*p* > 0.05) in single-dose glucose levels, long-term HDL levels, or body weights. However, glucose (mmol/L) levels in the four-week treated rats were considerably lower (16.2 ± 2.9, 21.6 ± 3.6, 86.8 ± 18.2, 29.8 ± 10.9; *p* > 0.05) than in the controls (21.63.6), and LDL levels in the treated group were significantly lower by 66% (*p* > 0.01; 86.818.2 against 29.810.9).	[111]
		*In vivo*	Kolaviron		At a dose of 100 mg kg^1^, kolaviron-linked biflavonoids effectively reduced hypoglycemic symptoms in normal and alloxan diabetic rabbits.	[112]
8	Analgesic	*In vivo*	Seeds	Ethanol	At all doses, there was a reduction in the number of writhes compared to control animals at *p* < 0.05. The seed has antianalgesic properties.	[27]
		*In vivo*	Seed		The findings reveal that the chemical has antinociceptive activity against acetic acid-induced abdominal constriction in mice in a dose-dependent manner.	[108]
9	Antipneumonia		Seeds		With a drop in the concentration of seeds, anti-*Klebsiella pneumonia* activity increased. The seed was effective at 500 mg/kg and exhibited significance at *p* < 0.0001.	[28]
10	Antiobesity	*In vivo*	Seeds	Ethanol	The results revealed a considerable rise in the counts of RBCS in both tested animals, as well as a reduction in their weight. Very low-level density lipoprotein in the plasma was reduced in the dependent-dose approach, while the level of chylomicrons increased in a dependent-dose approach. Low levels of high-density lipoproteins and an increase in low-density lipoproteins play a role in cardiovascular disease.	[113]
11	Fertility evaluation	*In vivo*	Seeds		For 28 days, animals were grouped into 4. 100, 200, and 400 mg/kg body extracts were given in groups 2, 3, and 4, respectively. A solution of normal saline was given to the control group. When comparing serum levels of LH and testosterone in rats treated with bitter kola extract to those in the control group, a dose-dependent drop was detected at *p* < 0.05. On the other hand, the extract did not affect FSH levels in the blood at any of the amounts tested. This finding revealed that bitter kola could lower fertility in male Wistar rats.	[33]
		*In vivo*		Ethanol	Experimental models were grouped into three: groups 1 and 2 were given extracts orally at doses of 400 and 200 mg for 28 days, respectively, while group 3 was the control. Group 1 exhibited modest interstitial congestion, disorientation of the cells, whereas group 2 had a normal interstitial space with the regeneration of the germinal epithelium and a small number of matured spermatozoa, according to the study. As a result, this research implies that excessive intake may have a more negative impact on sperm parameters and testis shape.	[32]
		I*n vivo*	Seed	Ethanol	The extract has been shown to have an antispermatogenic effect in male Wistar rats. It may be harmful to male reproductive health, necessitating managing its intake rate.	[114]
		*In vivo*	Seed	Aqueous	In a dose-dependent manner, the extract reduced sperm motility, concentration, and viability and affected normal sperm cell morphology.	[115]
		*In vitro*	Seeds	Methanol	The seeds extract had dose-dependent effects on induced cholinergic contractions and spasms generated by cumulatively raised concentrations of barium chloride and acetylcholine.	[116]
12	Antitrypanosoma	*In vivo*	Seeds	50 and 100% and methanol	Except for the group given 600 mg/kg body weight per day of 50% of the extract, which had a very low parasite count for nearly four months after treatment was terminated, but all treated died.	[35]
		*In vivo*	Seeds	Ethanol	This study found the extract and its alkaloid, flavonoid, and saponin fractions, at 50 and 100 mg/kg, have anti-*Trypanosoma brucei* activity.	[117]
	Anticancer		Seed/essential oil	n-hexane	At an 8.3 mg/mL dosage, this essential oil showed significant anticancer activity against MCF-7, A549, and Hela cell lines, with inhibition of 96, 0.9, 98, 0.5, and 94%, respectively.	[45]
13	Ingestion	*In vivo*	Seeds	Ethanol	The findings revealed erythrocyte count, PCV, and hemoglobin concentration values had all reduced dramatically. This demonstrates that it is an active ingredient and has no long-term toxicological implications when tested on mammalian erythrocytes.	[118]
14	Geotactic behavior	*In vivo*	Seeds		In flies fed a diet enriched with higher *G kola* seed inclusions, GST and catalase activities were dramatically boosted, whereas NO content was significantly reduced compared to controls.	[42]
15	Steroid hormones	*In vivo*	Seed	70% Ethanol	These findings suggest that they play a role in regulating cortisol, potassium, and sodium secretion control. Despite the possible benefits, it should be used with caution because it is a depressive.	[37]
16	Growth promoter	*In vivo*			Contain chemicals that reduce feed intake and growth performance. The effect appears to get stronger as the concentration gets higher. However, because the RBC and WBC levels increased, it is recommended that *G*. *kola* seeds be used in small doses or sporadically rather than continuously.	[119]
		*In vivo*	Seeds		The inclusion of *G*. *kola* in the feed resulted in the highest feed efficacy. The study found no evidence of mortality.	[120]
		*In vivo*	Seeds		There were no variations in the moisture, protein, or ash content of the fish carcasses between the treatments (*p* > 0.05). The findings imply that feeding *C*. *gariepinus* fingerlings *G*. *kola* seed powder increased growth rate, feed utilization, and survival.	[121]
		*In vivo*	Seed	Ethanol	The growth parameters and the food conversion ratio showed significant differences at *p* > 0.05. The fish fed a 1.0 g/kg ethanolic extract diet gained the most weight compared to the other treatments. This supports the probiotic benefits of the plant as a growth promoter.	[122]
		*In vivo*	Seeds		There are no differences in any performance indicators assessed between birds treated with BK 5 and those treated with BK 10. Birds on BK 5 showed greater FW, WG, and ADWG (*p* > 0.05). At a 5 g/kg diet, sun-dried ground bitter kola could be utilized as a supplement in broiler diets.	[123]
17	Liver injury	*In vivo*		70% ethanol	The findings showed that combining the two plants had a therapeutic effect on the wounded liver's repair. This supported its long-standing use in the treatment of liver-infected patients.	[124]
18	Haematological evaluation	*In vivo*	Seed	Aqueous	These data imply that it has no negative effects on the liver's activity and may have a favorable effect, as evidenced by its ability to reduce serum total cholesterol content and boost WBC count significantly.	[41]
		*In vivo*	Seeds	Ethanol	As a result, this extract has a minor erythropoietic impact, but a moderate leucopenia characterized by lymphocytosis but a decrease in all other WBC lines.	[125]
		*In vivo*	Seed		The result revealed that the meal increases the number of lymphocytes in rabbit bucks, which lead to an increase in total white blood cell count. Serum biochemical features revealed possible modest organ degeneration, as evidenced by a substantial (*p* > 0.05) increase in aspartate amino transaminase (AST) and alanine amino transaminase (ALT) in rats fed *Garcinia kola* seed meal diets.	[126]
		*In vivo*	Seed	Ethanol	The extract reduced the volume of the cell, mean cell, and hemoglobin cell mean in the animals' plasma substantially (*p* > 0.05). As a result of its antioxidant properties, the ethanolic extract of *G. kola* seed has hematological, stimulating, and boosting effects.	[40]
		*In vivo*	Seed	Ethanol	White blood cells proliferated significantly in this study, with a *p* > 0.05. Given the critical function that white blood cells play in the body's immune defense mechanism in organisms, this most likely explains the antibacterial activity of ethanolic extracts of plants.	[122]
19	Cytotoxicity	*In vivo*	Seeds		Compared to control flies, the high concentration of the plant in the diet dramatically reduced the survival rate of the experimental model. These findings could be linked to the bioactivity of *G*. *kola* seed compounds, including saponins and glycosides, which are hazardous at high concentrations. As a result of this research, it appears that excessive use of *G*. *kola* seeds may have toxicological consequences and that moderate consumption is therefore advised.	[42]
		Median lethal dose (LD50)	Stem bark	Methanol	The extract did not appear to have any significant toxicological effects on the erythrocytes, although it did exhibit a propensity to increase the number of erythrocytes over time.	[127]
		*In vivo*	Seed		CdCl_2_ dramatically reduced the number of spermatozoa in the seminiferous tubules, resulting in decreased spermatogenesis, sperm counts, and histopathology.	[128]
		*In vivo*	Seeds	Ethanol, aqueous	Compared to the control, the results demonstrated that neither of the medicinal plant extracts had any significant deleterious effects on total protein or glutamate pyruvic transaminase at *p* > 0.05.	[86]

*Note*. MIC: Minimum Inhibitory Concentration, MBC: Minimum Bacterial Concentration, DPPH: 2,2-diphenyl-1-picrylhydrazyl, FRAP: Ferric Reducing Antioxidant Power (FRAP) Assay.

## Data Availability

Data are available within the manuscript.
